# Tea *GOLDEN2*-*LIKE* genes enhance catechin biosynthesis through activating R2R3-MYB transcription factor

**DOI:** 10.1093/hr/uhac117

**Published:** 2022-05-17

**Authors:** Lihuan Wang, Xiaofeng Tang, Shiqiang Zhang, Xiang Xie, Mengfei Li, Yongsheng Liu, Songhu Wang

**Affiliations:** School of Horticulture, State Key Laboratory of Tea Plant Biology and Utilization, Anhui Agricultural University, Hefei 230036, China; School of Food and Biological Engineering, Hefei University of Technology, Hefei, 230009 China; School of Food and Biological Engineering, Hefei University of Technology, Hefei, 230009 China; School of Food and Biological Engineering, Hefei University of Technology, Hefei, 230009 China; School of Horticulture, State Key Laboratory of Tea Plant Biology and Utilization, Anhui Agricultural University, Hefei 230036, China; School of Horticulture, State Key Laboratory of Tea Plant Biology and Utilization, Anhui Agricultural University, Hefei 230036, China; Ministry of Education Key Laboratory for Bio-resource and Eco-environment, College of Life Science, State Key Laboratory of Hydraulics and Mountain River Engineering, Sichuan University, Chengdu, Sichuan 610064, China; School of Horticulture, State Key Laboratory of Tea Plant Biology and Utilization, Anhui Agricultural University, Hefei 230036, China

## Abstract

The biosynthesis of catechins, a major type of flavonoids accumulated in tea, is mediated by developmental cues and environmental stimuli. Light enhances but shading treatment reduces catechin accumulation in tea leaves. However, the transcription factors involved in light-mediated catechin biosynthesis remain to be identified. Two *GOLDEN2 LIKE* genes from tea plant (*CsGLK1* and *CsGLK2*) were isolated and characterized in both tomato and tea plants. Transcripts of both *CsGLK1* and *CsGLK2* were affected by light intensity in tea plants. Overexpression of *CsGLK1* and *CsGLK2* promoted chloroplast development and carotenoid accumulation in tomato fruits. An integrated metabolomic and transcriptomic approach revealed that both catechin content and related biosynthetic genes were upregulated in *CsGLK-*overexpressing tomato leaves. Our further studies in tea plants indicated that *CsGLK*s directly regulate the transcription of *CsMYB5b*, a transcription factor involved in catechin biosynthesis. Suppression of *CsGLK*s in tea leaves led to the reduction of both *CsMYB5b* expression and catechin accumulation. Taken together, the results show that *CsGLK*s are involved in light-regulated catechin accumulation in tea plants by regulating expression of *CsMYB5b* and have great potential for enhancing the accumulation of both carotenoids and flavonoids in fruits of horticultural crops.

## Introduction

Tea, made from tea plant (*Camellia sinensis*) leaves, is a popular non-alcoholic beverage worldwide and provides numerous benefits for human health [1–3], including its anti-cancer properties, which are mainly attributed to catechins [4–6]. Catechins are polyphenols of the flavan-3-ol type and are majorly accumulated in tea, usually accounting for >25% of tea leaf biomass [7]. Catechins are synthesized by the flavonoid pathway and some key synthetic enzymes, such as anthocyanidin reductase (ANR) and leucoanthocyanidin reductase (LAR), have been cloned and characterized in tea plants [8–15].

The MYB transcription factors, including AtPAP1, AtTT2, MtMYB14, and VvMYBPA1, play a predominant role in the accumulation of flavan-3-ols and proanthocyanidins (PAs), their polymers, in many plant species [16–18]. Their homologous *MYB* genes in tea plant include *CsMYB5b* and CsMYB75. CsMYB5b enhances the synthesis of both catechins and PAs in tobacco leaves by upregulating tobacco *LAR* and *ANR* [12, 14]. CsMYB75 is responsible for anthocyanin hyperaccumulation in purple tea [19]. In addition, catechins and PAs were accumulated by co-expressing an *Arabidopsis* PAP1 MYB transcription factor (AtPAP1) and a *Medicago* ANR (MtANR) in tobacco and *Medicago* [20]. Light plays an indispensable role in the biosynthesis of catechins and PAs [21–23]. However, the transcription factors involved in light-mediated catechin biosynthesis remain to be identified.

The Golden2-like (GLK) transcription factors are key regulators of chloroplast development in many plant species, including maize [24], *Arabidopsis* [25, 26], rice [27], moss [28], pepper [29], tomato [30, 31], and kiwifruit [32]. The flowering plants usually contain two *GLK* genes (*GLK1* and *GLK2*), due to a recent genome duplication [33]. Both *GLK1* and *GLK2* are expressed in photosynthetic tissue and exhibit functional redundancy [25, 31]. Knockout of both *GLK* genes in *Arabidopsis* resulted in reduced chlorophyll levels and chloroplast size and number, because of the impaired expression of genes involved in photosystems and chlorophyll biosynthesis [25, 26]. Overexpression of tomato *GLK2* (*SlGLK2*), whose mutation is responsible for the uniform ripening phenotype in fruits, increased the chloroplast levels and thereby enhanced the nutritional quality of fruits [30, 31] since the carotenoids are synthesized and stored in chromoplasts, which are converted from chloroplasts. More chromoplasts lead to greater accumulation of carotenoids [34, 35].

Light induces *GLK* expression, suggesting that *GLK*s are important for light-induced chloroplast development [25]. A recent study indicated that dark induces GLK1 degradation while light can stabilize and activate GLK1 by BIN2-mediated phosphorylation in *Arabidopsis* [36]. In tomato, SlGLK2 was degraded by the CUL4-DDB1-DET1 ubiquitin E3 ligase complex [37], which is a crucial component of light signaling and is required for plant photomorphogenesis [38]. Loss-of-function mutants of the E3 ligase complex displayed higher chloroplast levels and increased carotenoid accumulation in tomato fruits [34, 35, 39], possibly because SlGLK2 is stabilized in these mutants [37]. UV-B light increases *SlGLK2* expression and overexpression of the tomato UV-B receptor *UVR8* (*SlUVR8*) also enhances chloroplast development and carotenoid accumulation by increasing SlGLK2 accumulation [40].

The functions of *GLK* genes in chloroplast development and carotenoid accumulation were characterized previously. However, it remains to be determined whether *GLK* genes regulate flavonoid biosynthesis. In this study, we demonstrated that tea *GLK* genes (*CsGLK1* and *CsGLK2*) not only promote chloroplast development and carotenoid accumulation but also participate in the light-mediated biosynthesis of catechins and PAs.

## Results

### Protein alignment and subcellular localization

The tomato GLK2 protein sequence was used for BLAST in the tea genome sequence database (http://tpia.teaplant.org/index.html) [41]. Six loci were obtained and highlighted in the green frame in Supplementary Data Fig. S1. Only two loci (*TEA015144* and *TEA009544*) contain the complete nuclear localization signal (NLS, as indicated in the yellow frame in Supplementary Data Fig. S1), GARP DNA-binding domain (DBD, as indicated in the blue frame in Supplementary Data Fig. S1), and AREAEAA/AREVEAA hexapeptide (highlighted in the red frame in Supplementary Data Fig. S1). All these domains are conserved and essential for GLK functions [24]. For further functional characterization, these two genes were isolated and named *CsGLK2* and *CsGLK1*, respectively, based on protein sequence alignment (Supplementary Data Fig. S2A) and phylogenetic tree analysis (Supplementary Data Fig. S2B).

The protein sequence alignments (Supplementary Data Fig. S2A) showed that the CsGLKs have an N-terminal DBD domain (marked by a black line), and a conserved GOLDEN2 C-terminal (GCT) box [24]. In addition, the hexapeptide sequence (AREAEAA/AREVEAA, marked with a black asterisk) is also highly conserved in the GLK proteins [25]. The alignment analysis showed that CsGLKs contain typical domains that determine the GLK protein family. The phylogenetic tree analysis (Supplementary Data Fig. S2B) showed that CsGLK1 and CsGLK2 homologs were more closely related to kiwifruit AchGLK [32].

To examine the subcellular localization, *GFP* was fused to the C terminuses of *CsGLK1* and *CsGLK2*. Transient expression in tobacco protoplasts showed that CsGLK1/CsGLK2-GFP were localized in the nucleus, while the GFP (green fluorescent protein) signal was detected in the cytoplasm (Supplementary Data Fig. S3). These results confirmed the nuclear localization of both CsGLK1 and CsGLK2.

### Expression pattern of *CsGLK1* and *CsGLK2*

**Figure 1:**
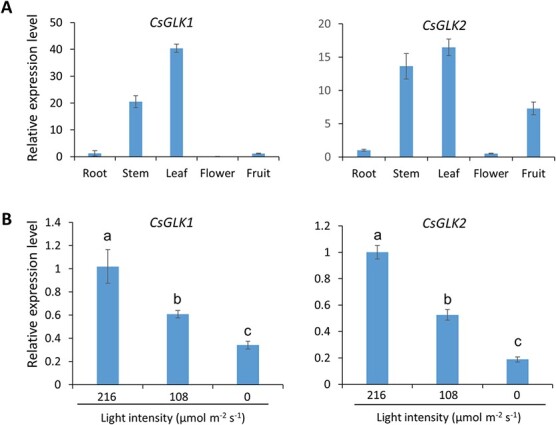
Expression patterns of *CsGLK1* and *CsGLK2* in tea plants. (A) Expression of *CsGLK1* and *CsGLK2* in root, stem, leaf, flower, and fruit of local cultivar ‘Shuchazao’ determined by qRT–PCR analysis. (B) Expression levels of *CsGLK1* and *CsGLK2* in plants cultured in control light (light intensity 216 μmol m^−2^ s^−1^), shading (108 μmol m^−2^ s^−1^), and dark (0 μmol m^−2^ s^−1^) conditions for 24 hours. Error bars represent standard deviations of three biological replicates. Different letters above the columns indicate statistically significant differences (Duncan test, *P* < .05).

To study the tissue-specific expressions of *CsGLK1* and *CsGLK2*, total RNAs were extracted from different tissues of tea plants. qRT–PCR assays showed that *CsGLK*s were expressed in the leaves and stems tested, but at a very low level in roots and flowers (Fig. 1A). *CsGLK2* showed much higher expression in tea fruits than *CsGLK1* (Fig. 1A), similar to the patterns of *SlGLK*s in tomato [30]. Besides, both genes were downregulated by treatment with decreased light intensity (Fig. 1B). Only 34% of *CsGLK1* transcripts and 18% of CsGLK2 transcripts were detected in the dark, compared with control light (216 μmol m^−2^ s^−1^) (Fig. 1B). These results indicated that light regulates the expression of *CsGLK*s in tea plants.

### *CsGLK* overexpression enhanced chloroplast development and carotenoid accumulation in tomato

**Figure 2:**
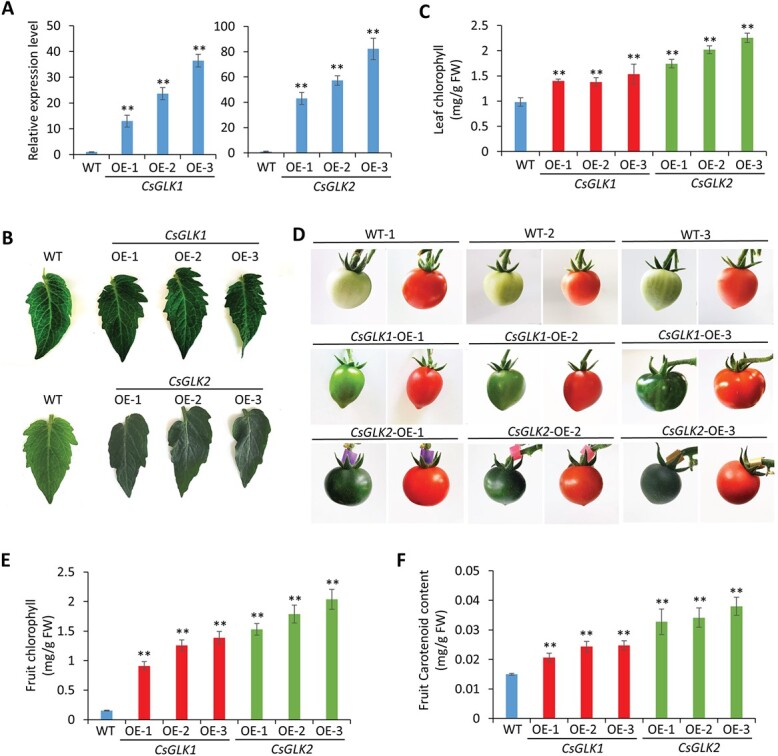
Characterization of tomato plants overexpressing *CsGLK1* and *CsGLK2*. (A) qRT–PCR analysis of *CsGLK1* and *CsGLK2* expression in three independent transgenic ‘Micro-Tom’ lines overexpressing *CsGLK1* (*CsGLK1* OE-1, -2, and -3) and *CsGLK2* (*CsGLK2* OE-1, -2, and -3). Error bars indicate standard deviation of three biological replicates. (B) Leaf morphology of 40-day-old WT and transgenic *CsGLK1* OE and *CsGLK2* OE plants. (C) Total chlorophyll contents in leaves from 40-day-old WT and transgenic plants shown in (B). FW, fresh weight. (D) Fruits in mature green and red ripe stage of WT and three transgenic lines of *CsGLK1* OE and *CsGLK2* OE. (E, F) Total chlorophyll contents in mature green fruits (E) and carotenoid contents in red ripe fruits (F) of WT and transgenic plants. Mean ± standard deviation values were obtained from at least 15 measurements. ^**^P < .001 (Student’s *t*-test).

The ‘Micro-Tom’ tomato is suitable for functional verification of *GLK*s because it contains an insertion mutant in the *SlGLK2* gene, which causes the uniform ripening phenotype in fruits [30]. Therefore, we used the CaMV35S promoter and ectopically overexpressed *CsGLK1* and *CsGLK2* in ‘Micro-Tom’ tomato plants to investigate their function in the regulation of chloroplast development. More than 10 transgenic tomato lines were obtained. For each construct, three independent transgenic lines (CsGLK1-OE-1, -2, and -3; CsGLK2-OE-1, -2, and -3) with the highest expression were chosen for further analysis and their transcription levels were verified by qRT–PCR (Fig. 2A). As expected, all transgenic plants overexpressing *CsGLK1* or *CsGLK2* displayed darker green leaves (Fig. 2B) and immature fruits (Fig. 2D) than wild-type (WT) plants. Consistently, increased chlorophyll contents in leaves (Fig. 2C) and fruits (Fig. 2E) were detected in the *CsGLK*-overexpressing lines. Transmission electron microscopy (TEM) observation showed a significant increase in chloroplast number per square millimeter in sections of fruits (Supplementary Data Fig. S4A and B) and leaves (Supplementary Data Fig. S4C and D) from *CsGLK*-overexpressing lines compared with WT. *CsGLK1* or *CsGLK2* overexpression also resulted in significantly increased chloroplast size, as indicated by the increased chloroplast areas in the transgenic fruits (Supplementary Data Fig. S4A and B) and leaves (Supplementary Data Fig. S4C and D). Similar phenotypes were observed in tomato fruits overexpressing *SlGLK2* [30, 31] or *AchGLK* [32], suggesting that *CsGLK*s share the conserved function in promoting chloroplast development and chlorophyll synthesis.

Previous studies showed that the carotenoid content of fruit is positively correlated with chloroplast development [31, 34]. Therefore, we also analyzed carotenoid accumulation in the fully ripened fruits of *CsGLK1/CsGLK2*-overexpressing lines, and the results indicated that total carotenoid contents were obviously increased in the transgenic lines (Fig. 2F).

### Metabolomic and transcriptomic analysis of *CsGLK*-overexpressing leaves

**Figure 3:**
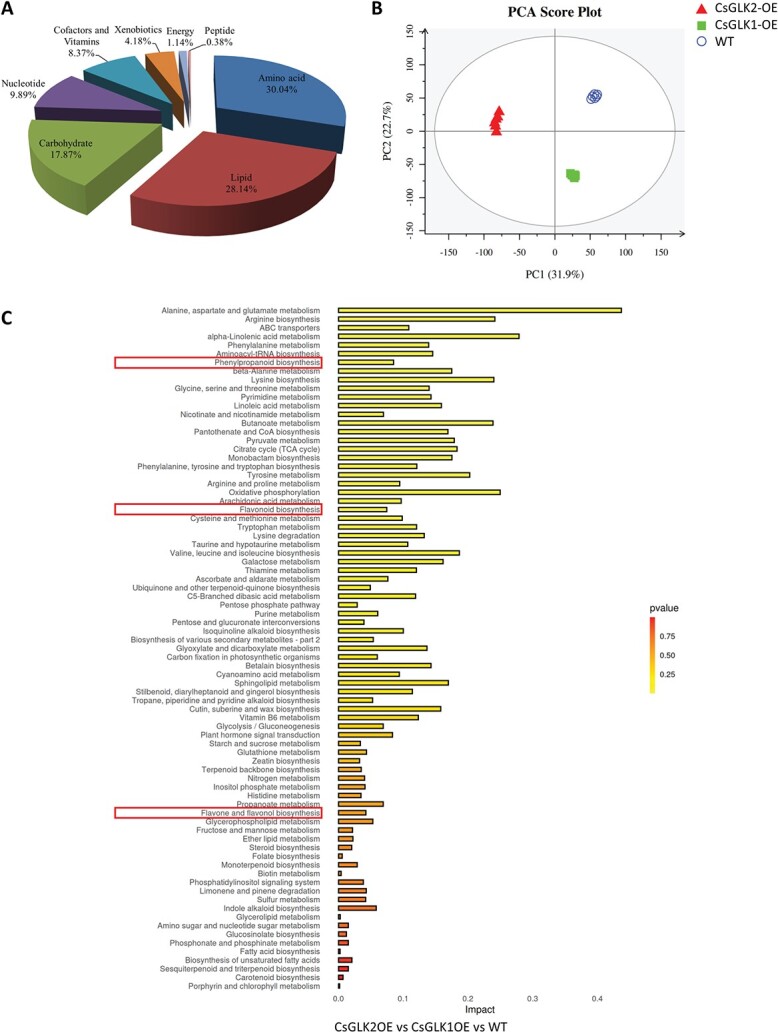
Metabolomic analysis of tomato leaves overexpressing *CsGLK1* and *CsGLK2*. (A) Identified metabolites were classified into several categories, including amino acids, lipids, carbohydrates, nucleotides, cofactors and vitamins, xenobiotics, energy, and peptides. According to their percentages with respect to total metabolites, the top 10 categories were selected to draw the pie chart. (B) PCA of all samples from three different genotypes. Red triangles indicate *CsGLK2*-OE plants. Green squares indicate *CsGLK1*-OE plants. Blue circles indicate WT plants. (C) KEGG analysis of DAMs among three genotypes (*CsGLK2-*OE versus *CsGLK1-*OE versus WT). The red frames highlight the pathways related to flavonoid biosynthesis.

Since tea is made from the leaves of tea plants and it remains largely unknown how GLK influences leaf metabolism, we used metabolomic assay to investigate the alterations of leaf metabolism in *CsGLK*-overexpressing tomato plants. Six biological replicates were analyzed for WT and *CsGLK1*- and *CsGLK2*-overexpressing plants. Each replicate was actually a pooled sample collected from three independent transgenic lines or WT individuals. A total of 297 annotated metabolites were identified in at least one of the samples (Supplementary Data Table S1), including ~30% amino acids, 28% lipids, 18% carbohydrates, 10% nucleotides, 8% cofactors and vitamins, 4% xenobiotics, and 2% other groups (Fig. 3A). All identified metabolites were analyzed by agglomerate hierarchical clustering and are presented in a heat map (Supplementary Data Fig. S5). Principal component analysis (PCA) showed that these samples could be divided into three groups for WT, *CsGLK1*-, and *CsGLK2*-overexpressing plants, respectively, indicating the reliable repeatability of these samples derived from three different genotypes (Fig. 3B).

Next, more than 160 differently accumulated metabolites (DAMs) were identified among three genotypes (Supplementary Data Table S1). KEGG (Kyoto Encyclopedia of Genes and Genomes) pathway analysis indicated that these DAMs were obviously enriched in the processes of primary metabolism, such as the metabolisms of amino acids, ABC transporters, and energy, as well as in some secondary metabolisms, such as α-linolenic acid metabolism and isoquinoline alkaloid biosynthesis (Fig. 3C). Among them, phenylpropanoid, flavonoid, flavone, and flavonol biosyntheses were highly related (highlighted by red frames in Fig. 3C). These results indicated that *CsGLK*s are involved not only in primary metabolisms but also in some secondary ones, especially flavonoid biosynthesis, which is pivotal for the nutrient qualities of tea.

**Figure 4:**
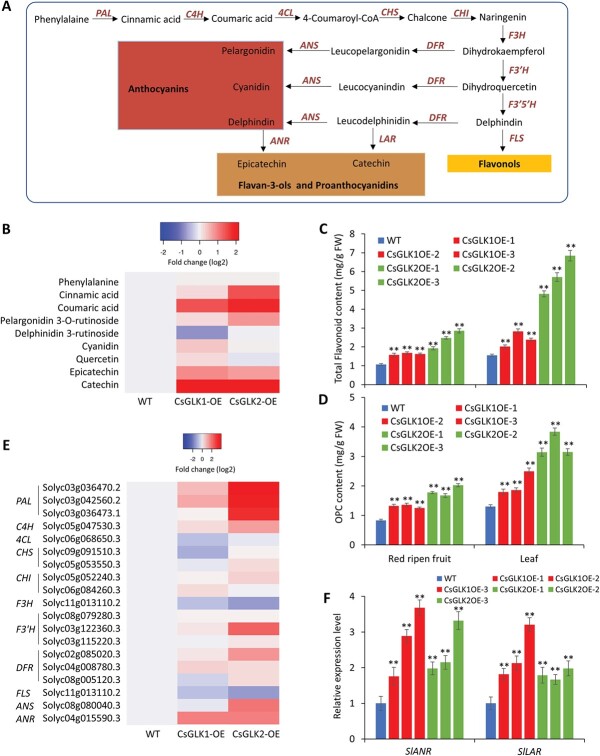
*CsGLK* overexpression enhanced flavonoid biosynthesis. (A) Schematic diagram of the flavonoid biosynthesis pathway with the catalytic enzymes. (B) The heat map shows the contents of detected flavonoid metabolites in WT and transgenic plants (*CsGLK1*-OE and *CsGLK2*-OE). Data represent average values from six biological replicates. (C, D) Total flavonoid (C) and OPC (D) contents in leaves and red ripe fruit of WT and transgenic lines. FW, fresh weight. Mean ± standard deviation values were obtained from at least 15 measurements. ^**^P < .001 (Student’s *t*-test). (E) Heat map of relative expression levels of flavonoid biosynthetic genes identified in the transcriptome analysis of three genotypes (WT, *CsGLK1*-OE, and *CsGLK2*-OE). Data represent average values of three biological replicates. (F) qRT–PCR analysis of *SlANR* (Solyc04g015590) and *SlLAR* (Solyc05g009860) in WT and transgenic lines. Error bars indicate standard deviation of three biological replicates.

To further investigate how *CsGLK*s affect flavonoid biosynthesis (Fig. 4A), we presented the flavonoid-related metabolites detected in the metabolomic assays as the heat map shown in Fig. 4B. Among these metabolites, cinnamic acid, coumaric acid, epicatechin, and catechin were significantly increased in both the *CsGLK1*- and *CsGLK2*-overexpressing lines compared with WT plants (Fig. 4B). Catechin was increased by 4.49 and 4.05 times in the *CsGLK1*- and *CsGLK2*-overexpressing lines, respectively (Supplementary Data Table S1). To further confirm these results, we used two commercial assay kits to quantitatively measure the total flavonoids (Fig. 4C) and oligomeric proanthocyanidin (OPC) contents (Fig. 4D), in both transgenic leaves and fruits. HPLC analysis of catechin monomers (Supplementary Data Fig. S6) also confirmed that catechin (C) and epicatechin gallate (GCG) are substantially accumulated in the transgenic tomato leaves. The results showed that *CsGLK* overexpression enhanced flavonoid and OPC accumulation in both fruits and leaves.

Next, we performed transcriptome analysis for the same samples as those used in the metabolomic analysis and all the differentially expressed genes (DEGs) are listed in Supplementary Data Table S2. Most of the flavonoid biosynthetic genes (Fig. 4A) were detected in our transcriptome and the average levels of their transcripts in three replicates are shown in a heat map in Fig. 4E. The *PAL*, *C4H*, *F3′H*, *DFR*, and *ANR* genes were increased in both *CsGLK1*- and *CsGLK2*-overexpressing lines (Fig. 4E). Most of their expression patterns were validated by qRT–PCR (Supplementary Data Fig. S7). The enhanced expression of *PAL*s and *C4H* might be responsible for the increased accumulation of cinnamic and coumaric acids. Upregulation of *F3′H*, *DFR*s, and *ANR* genes (Fig. 4E) should contribute to the increased accumulation of epicatechin and catechin (Fig. 4B). Consistent with the transcriptome analysis, an increase in *ANR* expression was detected in the *CsGLK1*- and *CsGLK2*-overexpressing lines by qRT–PCR assays (Fig. 4F). Although the *LAR* gene was not identified in our transcriptomic analysis, our qRT–PCR showed that it was also upregulated in both transgenic lines (Fig. 4F). These results indicated that the enhanced catechin and epicatechin accumulation possibly resulted from upregulation of some flavonoid biosynthetic genes, including *SlLAR* and *SlANR*, which play important roles in catechin and epicatechin biosynthesis.

### CsGLKs are involved in flavonoid metabolism by directly activating transcription of *CsMYB5b* in tea plant

**Figure 5:**
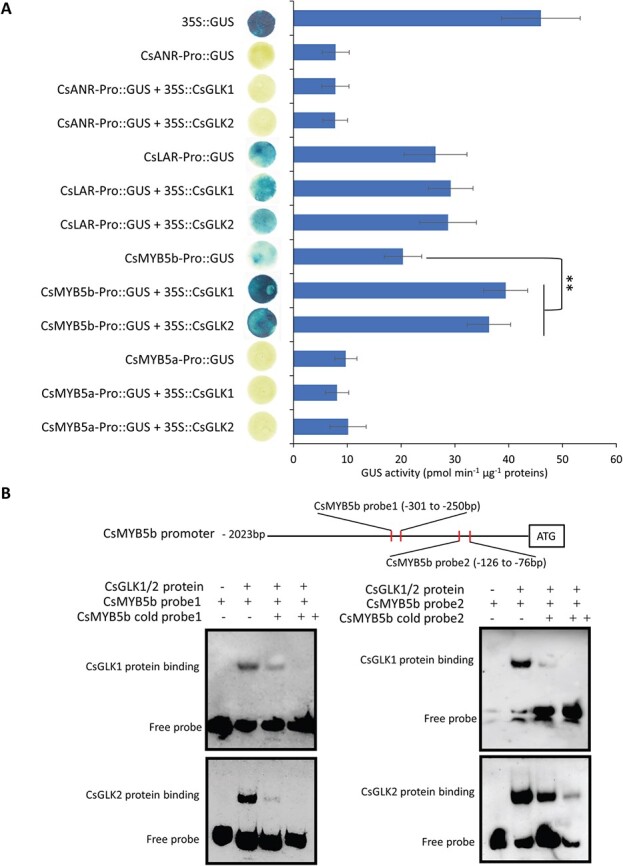
CsGLKs activate the promoter of *CsMYB5b* but not that of structural genes in catechin biosynthesis. (A) GUS staining and activities detected in *N. tabacum* leaves transiently expressing *CsANR-Pro*::*GUS*, *CsLAR-Pro*::*GUS*, *CsMYB5b-Pro*::*GUS*, *CsMYB5a-Pro*::*GUS*, or 35S::CsGLK1/CsGLK2 combined (shown by ‘+’) with these reporter constructs. 35S::*GUS* was used here as a positive control. Circle leaves with blue or yellow color represent GUS staining. GUS activities were measured quantitatively and are shown as means ± standard deviation of six independent assays. ^**^P < .001 (Student’s *t*-test). (B) EMSA between CsGLK1/2 and the promoters of *CsMYB5b*. Purified His-tagged CsGLK1 and CsGLK2 proteins were incubated with unlabeled probe (cold) or biotin-labeled probe, and DNA–protein complexes were separated on native polyacrylamide gels, then photographed. The presence or absence of specific probes is marked by the symbol ‘+’ or ‘−’. Specific binding of CsGLKs to promoters of *CsMYB5b* probe 1 (left) and *CsMYB5b* probe 2 (right).

To check whether CsGLKs directly activate *LAR* and *ANR* in tea plants, the native promoters of *CsLAR* (*CsLAR-Pro*) and *CsANR* (*CsANR-Pro*) were cloned from the genome of ‘Shuchazao’ and fused with the reporter gene *GUS* to construct *CsLAR-Pro::GUS* and *CsANR-Pro::GUS*, respectively. The transient expression of *CsANR-Pro::GUS* produced very low GUS activity compared with that of *35S::GUS* and the co-expressions of *CsANR-Pro::GUS* with *35S::CsGLK1* (or *35S::CsGLK2*) had no obvious effect on GUS activity (Fig. 5A). Similarly, the combined expression of *CsLAR-Pro::GUS* with *35S::CsGLK1* (or *35S::CsGLK2*) did not produce higher GUS activity than the individual expression of *CsLAR-Pro::GUS* (Fig. 5A). These results indicated that CsGLKs cannot activate the promoters of the *CsLAR* or *CsANR* genes.

Among the DEGs from our transcriptomic analysis, several MYB transcription factors were identified (Supplementary Data Table S2 and Supplementary Data Fig. S8), including *SlMYB54*, the highly homozygous gene of *CsMYB5b* that was demonstrated to positively regulate catechin biosynthesis by activating *LAR*s and *ANR*s in tea plants [12, 14]. The qRT–PCR analysis confirmed that *SlMYB54* and other identified *MYB* genes were indeed upregulated in the *CsGLK1*- and *CsGLK2*-overexpressing transgenic tomato lines (Supplementary Data Fig. S8), suggesting that CsGLKs might enhance catechin synthesis by directly activating the transcription of *CsMYB5b* in tea plants. To support this notion, we expressed *CsMYB5b-Pro::GUS* alone or co-expressed with *35S::CsGLK1* (or *35S::CsGLK2*) in tobacco leaves. We observed significantly higher GUS activities in the co-expressed combinations than with the expression of *CsMYB5b-Pro::GUS* alone (Fig. 5A). In addition, the native promoter of another *MYB* gene (*CsMYB5a*), which promotes the accumulation of PAs [42], was also cloned and fused with *GUS*. The combined expression of *CsMYB5a-Pro::GUS* and *35S::CsGLK1* (or *35S::CsGLK2*) did not alter the GUS activities compared with expression of *CsMYB5a-Pro::GUS* alone (Fig. 5A). These results suggest that CsGLKs involve transcriptional activation of *CsMYB5b* but not *CsMYB5a*. Next, the electrophoretic mobility shift assay (EMSA) was used to study whether CsGLKs can physically bind the promoter of *CsMYB5b*. Based on the identified *cis*-elements that GLK binds in *Arabidopsis* [26, 36], we found three similar element motifs in the promoter of *CsMYB5b* and designed three corresponding probes (probes 1, 2, and 3) (Supplementary Data Fig. S9A). EMSA results showed that CsGLKs were capable of binding probes 1 and 2 *in vitro* (Fig. 5B), but not probe 3 (Supplementary Data Fig. S9B). These results indicated the direct interaction between CsGLKs and the promoter of *CsMYB5b*.

**Figure 6:**
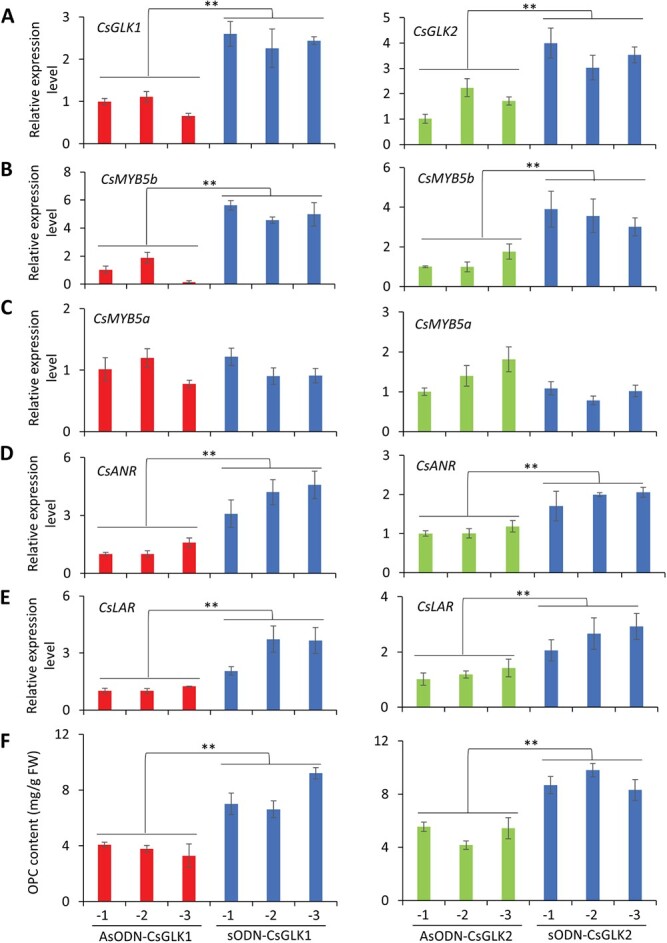
*CsGLK* silencing impaired *CsMYB5b* expression and catechin biosynthesis in tea plants. (A–E) qRT–PCR analysis of mRNA levels of *CsGLK1* and *CsGLK2* (A), *CsMYB5b* (B), *CsMYB5a* (C), *CsANR* (D), and *CsLAR* (E) in tea plants that were treated with antisense oligonucleotides (AsODNs) and sense oligonucleotides (sODNs) against *CsGLK1* and *CsGLK2*. Mean ± standard deviation values were obtained from six independent assays. (F) OPC contents of tea leaves treated with AsODN or sODN against *CsGLK1* and *CsGLK2*. FW, fresh weight. Mean ± standard deviation values were obtained from six independent assays. ^**^P < .001 (Student’s *t*-test).

To further confirm the involvement of CsGLK-mediated regulation of *CsMYB5b* expression and catechin biosynthesis, a gene-specific antisense oligonucleotide (AsODN) method was employed to suppress the expression of *CsGLK1* and *CsGLK2* in *C. sinensis* leaves [43]. Consequently, the expression of *CsGLK1* in tea leaves treated with AsODN_CsGLK1 was obviously decreased in three independent tests, compared with leaves treated with sense oligonucleotides of *CsGLK1* (sODN_CsGLK1) (Fig. 6A). The mRNA levels of *CsMYB5b* (Fig. 6B), *CsANR* (Fig. 6D), and *CsLAR* (Fig. 6E) and OPC contents (Fig. 6F) of the tea leaves were all significantly reduced in the AsODN_CsGLK1 plants compared with those in control (sODN_CsGLK1) plants. Similarly, *CsGLK2* silencing (Fig. 6A) also impaired the expressions of these genes (Fig. 6B, D, and E) and OPC contents (Fig. 6F). HPLC analysis of catechin monomers also confirmed that all the detectable monomers were significantly less in AsOND leaves than in sODN leaves (Supplementary Data Fig. S10). Consistent with the results of the above-mentioned GUS assays, suppression of *CsGLK*s had no obvious effect on *CsMYB5a* expression (Fig. 6C). These results confirmed that CsGLKs mediate catechin biosynthesis in tea leaves by transcriptionally regulating *CsMYB5b* but not *CsMYB5a*. Besides, light intensity also affects expression levels of *CsMYB5b*, *CsANR*, and *CsLAR* in the tea plant (Supplementary Data Fig. S11), which is consistent with the expression pattern of *CsGLK*s (Fig. 1B).

## Discussion

GLKs have been demonstrated to play a key role in chloroplast development in many plant species [25, 26, 30, 31]. Our results indicate that *CsGLK1* and *CsGLK2* from the tea plant also possess the conserved function in enhancing chloroplast development, as indicated by the increased chloroplast number and size in fruits and leaves of *CsGLK*-overexpressing tomato plants (Supplementary Data Fig. S4). The carotenoids, including lycopene, are very important nutrients of tomato fruits and are synthesized and accumulated in chromoplasts. *CsGLK*-overexpressing tomato fruits exhibit higher contents of carotenoids (Fig. 2) since they contain more chromoplasts than WT plants.

In most tomato cultivars, flavonoids are hardly produced in the fruits because of low expression of flavonoid biosynthetic genes in fruits [44, 45]. Flavonoids, a large family of polyphenolic compounds, include anthocyanins, flavan-3-ols, flavonols, flavones, flavanones, and isoflavones [46]. Overexpression of the petunia *CHI* gene increased flavonol levels in tomato fruits [44]. Mutation [47] or fruit-specific silencing [48] of the tomato *DET1* gene led to higher contents of flavonol (quercetin) and flavanone (naringenin) in fruits. Fruit-specific co-expression of a bHLH transcription factor, Del, and an MYB-related transcription factor, Ros1, resulted in purple tomato fruits with enhanced accumulation of anthocyanins [45]. Recent studies indicated that SlAN2-like (an MYB transcription factor) also regulates anthocyanin accumulation in both peel and flesh of tomato fruits [49, 50]. To our best knowledge, enhanced flavan-3-ol biosynthesis in tomato fruits has not been accomplished yet. Our work indicated that overexpression of *CsGLK*s enhanced the accumulation of flavan-3-ols, including catechin, epicatechins, and their polymers (PAs) in both leaves and fruits (Fig. 4).

The biosynthesis of catechin, a major type of flavonoid accumulated in tea, is mediated by both developmental cues and environmental stimuli [23, 51]. In most cases, light promotes catechin biosynthesis [21, 52, 53] and shading treatment reduces catechin accumulation in tea leaves [22, 23, 51, 54]. However, it remains largely unknown how light regulates catechin biosynthesis. Our results indicated that the transcription of both *GLK* genes was affected by various light intensities (Fig. 1B) and overexpression of *CsGLK*s significantly increased the contents of catechin and epicatechin (Fig. 4; Supplementary Data Fig. S6). Suppression of *CsGLK1* or *CsGLK2* expression in tea leaves resulted in reduced accumulation of catechin monomers (Supplementary Data Fig. S10) and OPC contents (Fig. 6). Interestingly, *CsGLK*-overexpressing tomato leaves substantially accumulated catechin and epicatechin gallate (Supplementary Data Fig. S10), indicating that tomato might have all the structural genes required for catechin biosynthesis and have great potentials for studying catechin accumulation. Besides, these results suggested that *CsGLK*s are involved in light-mediated catechin accumulation in tea plants and shading treatment decreases catechin contents, possibly through suppressing expression levels of *GsGLK*s (Supplementary Data Fig. S12). In *Arabidopsis*, *GLK* genes were also induced by light [25] and GLK proteins were degraded in the dark [36]. It appears that light-mediated *GLK* expression is also conserved in tea plants.

PAL catalyzes the rate-determining step in phenylpropanoid synthesis [55]. Previous studies indicated that both gene expression and enzyme activity of PAL are affected by shading treatment and *PAL* is involved in light-mediated catechin biosynthesis [21–23]. In *CsGLK*-overexpressing plants, transcripts of *PAL*s and the catalytic product cinnamic acid were significantly increased (Fig. 4). Besides, transcripts of *C4H*, *F3′H*, *ANR*, and *LAR* were decreased by shading treatment [23] and all were increased in *CsGLK*-overexpressing plants in our study (Fig. 4). These results indicate that CsGLKs participate in light-mediated activation of flavonoid biosynthetic genes.

LARs catalyze the leucoanthocyanidins to catechins [56] and ANRs convert anthocyanins into epicatechins [57]. Co-expression of *LAR* and *ANR* enhances the accumulation of flavan-3-ols and their polymeric PAs [12]. Although upregulation of *LAR* and *ANR* was observed in the *CsGLK*-overexpressing tomato lines, CsGLKs did not directly activate the promoters of *CsLAR* and *CsANR* (Fig. 5). Previous studies in many plant species indicated that MYBs, such as AtTT2, VvMYBPA1, and MtMYB14, mediate the biosynthesis of flavan-3-ols and PAs [16–18]. Their homologous *MYB* gene in the tea plant (*CsMYB5b*) was also proved to promote accumulation of flavan-3-ols and PAs by upregulating *LAR* and *ANR* [12, 14]. Our transcriptomic results revealed that several *MYB* genes, including *SlMYB54*, the homologous gene of *CsMYB5b* in tomato, were upregulated in the *CsGLK*-overexpressing plants (Supplementary Data Fig. S7). Therefore, we speculate that CsGLKs increased the transcripts of *LAR* and *ANR* genes, possibly through directly activating transcription of *CsMYB5b* in tea plants. Our further analysis confirmed that both CsGLKs recognized the promoter of *CsMYB5b* (Fig. 5). The AsODN-mediated suppression of *CsGLK*s in tea plant leaves also led to decreased expression of *CsMYB5b* and reduced accumulation of PAs (Fig. 6B and F). These results indicated that CsGLKs enhance catechin biosynthesis by directly regulating *CsMYB5b* transcription in tea leaves (Supplementary Data Fig. S12).

Metabolic activities in chloroplasts are highly oxidizing and the rapid electron flow frequently results in the production of chloroplastic reactive oxygen species (ROS) [58, 59]. The presence of ROS-generating centers makes the chloroplast a major organelle of ROS production, especially under fluctuating light intensity [60, 61]. Flavonoids, including catechin, are natural antioxidants and act as scavengers of ROS [62, 63]. On one hand, CsGLKs promote chloroplast development. On the other hand, they also enhance the biosynthesis of flavonoids, possibly to alleviate the oxidative stress caused by more chloroplasts under fluctuating irradiation. This speculation is consistent with a previous observation indicating that light-sensitive tea leaves produced large amounts of flavonoids, including catechin and epicatechin, which function in photoprotection and facilitate the acclimatization of tea plants by scavenging ROS [51]. Of course, this requires further investigations to demonstrate the coordination between CsGLK-enhanced chloroplast development and flavonoid biosynthesis.

Our characterization of tea *GLK* genes (*CsGLK1* and *CsGLK2*) has indicated that *CsGLK*s are involved in both chloroplast development and catechin accumulation in tea plants. These two genes are also promising candidates for genetic manipulation to enhance accumulation of both carotenoids and flavan-3-ols in fruits of horticultural crops.

## Materials and methods

### Plant material and growth conditions

Tea plants (*C. sinensis* ‘Shuchazao’) were grown in a tea plantation at Anhui Agricultural University, Hefei, China (117.27E, 31.86N). Roots, stems, mature leaves, flowers, and fruits of tea were collected and immediately frozen in liquid nitrogen. Shade treatment was performed using shading nets with a transmittance rate of 50% and the whole tea plants were covered by the shading nets for 24 hours. Control and shaded plants were grown in an incubator at 24°C with continuous light (light intensity 216 μmol m^−2^ s^−1^) for 24 hours. Dark treatment was performed in the same incubator without light.

WT tomatoes (*Solanum lycopersicum* ‘Micro-Tom’) were used in our laboratory. They were grown in a constant-temperature cultivation chamber at 24°C with a photoperiod of 16 hours light/8 hours dark.

Tobacco (*Nicotiana tabacum* and *N. benthamiana*) plants, which were used for GUS activation and subcellular localization, respectively, were cultivated in a constant temperature of 24°C and a photoperiod of 16 hours light/8 hours dark.

### Bacterial strains

*Escherichia coli* Trans-T1 was purchased from TransGen Biotech, Beijing, China. *Agrobacterium tumefaciens* EHA105 and GV3101 (Tolobio, Shanghai, China) were used for stable and transient expression, respectively.

### Cloning of *CsGLK1* and *CsGLK2*

Total RNA was extracted from the tea plants using an RNA Extraction Kit (Biofit, Beijing, China) according to the manufacturer’s instructions. Reverse transcription reactions were performed using the HiScript II 1st Strand cDNA Synthesis Kit (Vazyme, Nanjing, China). The gene was amplified with PrimeSTAR^®^ Max DNA Polymerase (Takara, Dalian, China) using cDNA. The PCR products were purified with a Gel Extraction Kit (Vazyme, Nanjing, China), ligated into a pEasy-Blunt Simple vector (TransGen Biotech, Beijing, China), and subsequently transformed into Trans-T1-competent cells for sequencing. The primers of *CsGLK1* (accession number MZ093621) and *CsGLK2* (MZ093620) are listed in Supplementary Data Table S3.

### Phylogenetic analysis of GLKs

The protein sequences of GLKs from kiwifruit, rice, tomato, and *Arabidopsis* were used. The tree was constructed according to the alignments, using MEGA 7.0 with the neighbor-joining method under the standard parameters. Alignment of amino acid sequences was conducted using DNAMAN 8.0.

### Quantitative real-time PCR

ChamQ™ Universal SYBR^®^ qPCR Master Mix was used based on the manufacturer’s instructions (Vazyme, Nanjing, China). The PCR reactions were performed in a CFX Connect™ Real-Time System (Bio-Rad, USA). Relative expression values were determined using the 2^−ΔΔCT^ method [64]. In the qRT–PCR analysis, error bars represent variation from three replicates for each sample; each sample was quantified in triplicate. Statistical significance was determined by Student’s *t*-test. All data were analyzed busing SPSS statistical software. The qRT–PCR primers are listed in Supplementary Data Table S3.

### Subcellular localization

The coding sequences of *CsGLK1* and *CsGLK2* were amplified by PCR with gene-specific primers (Supplementary Data Table S3). The product was inserted into vector pART27 containing the CaMV35S promoter and a GFP. The recombinant vectors 35S::*CsGLK1*/*CsGLK2*-*GFP* were transformed into *A. tumefaciens* strain GV3101, which was infiltrated into tobacco (*N. benthamiana*) leaves. The infiltrated plants were cultured for 2 days. Protoplasts from the injection site were isolated by enzymolysis (Cellulase R-10 and Macerozyme R-10, Yakult, Japan) and subsequently treated with 10 μg/mL DAPI for 30 min. The GFP signal was detected with a confocal microscope (FV1000; Olympus, Tokyo, Japan).

### Tomato transformation

Whole coding sequences for *CsGLK1* and *CsGLK2* were amplified and ligated into plant expression vector pBI121 containing the 35S promoter. The recombinant vectors 35S::*CsGLK1/CsGLK2*OE were transformed into *A. tumefaciens* strain EHA105. *A. tumefaciens*-mediated transformation was used to obtain transgenic plants according to the described procedure [34].

### Transmission electron microscopy analyses of chloroplast cells

For analysis by TEM, the pericarps of mature green fruits (20 days after anthesis) and leaves from transgenic and WT tomato plants were cut and fixed in electron microscope fixative (Servicebio, Wuhan, China). The sections were analyzed using a transmission electron microscope (Hitachi, HT7700). The chloroplast area was measured using Image J software.

### Determination of chlorophyll, carotenoid, total flavonoid, and OPC contents

Mature leaves from 40-day-old WT and transgenic plants and mature green fruits were harvested for total chlorophyll measurements, which were performed using the commercial Chlorophyll Assay Kit (Solabio, Beijing, China). Mature leaves and red ripe fruits were harvested for quantitative measurements of flavonoids, OPC contents, and carotenoids. These measurements were performed using the commercial Plant Flavonoids Assay Kit (Solabio, Beijing, China), the OPC Content Assay Kit (Boxbio, Nanjing, China), and the Plant Carotenoid Assay Kit (Solabio, Beijing, China), respectively.

### Metabolomic and transcriptomic analysis

Leaf tissues (six biological replicates, each of which was a pooled sample from three independent transgenic lines) of 40-day-old transgenic lines (CsGLK1OE and CsGLK2OE) and WT plants were harvested and examined by ultra-high-performance liquid chromatography–tandem mass spectrometry (UPLC–MS/MS). All metabolites were analyzed by comparison of the ion features in the samples according to a library of chemical standard entries, including molecular weight, preferred adducts, retention time, and in-source fragments as well as associated MS spectra and the results were checked for accuracy by visual inspection for quality control [65].

RNA-Seq cDNA libraries were obtained from RNA isolated from the above-mentioned tomato leaves (three biological replicates for each genotype) using the NEBNext mRNA Library for Illumina, and subsequently PCR-amplified using NEBNext Multiplex Oligos for Illumina (New England Biolabs, USA). A High Sensitivity DNA chip on a 2100 Bioanalyzer (Agilent Technologies, USA) was used to determine the quality and average length of cDNAs in the library. RNA-Seq libraries were sequenced on a HiSeq 2500 (Illumina) system according to the manufacturer’s instructions. DEG data were analyzed using methods similar to those described previously [66]. Genes with a fold change ≥2 and *P*-value < .01 were determined to be differentially expressed.

### Determination of catechin composition by reverse-phase HPLC

The HPLC assay was employed to determine the catechin monomers in tea and tomato leaves. One gram of powder was dissolved with 10 ml of 95% ethanol, purified and extracted with chloroform and ethyl acetate. After drying, the preparation was dissolved and the volume was fixed with 1 mL of methanol. The contents of filtered extracts were separated and detected by the HPLC assay using a C18 Aqua column (4.6 mm × 150 mm, i.d. 5 μm), a 1525 Binary Pump, and a 2489 UV/Visible Detector (Waters, USA). The injection volume was 10 μL. The mobile phase was 10% aqueous acetic acid (v/v) and 100% acetonitrile. Catechins were monitored at 280 nm. Chromatographic peaks were identified and quantified by comparison with six standards (Epigallocatechin/EGC, 970-74-1; Catechin/C, 154-23-4; Epicatechin/EC, 490-46-0; Epigallocatechin gallate/EGCG, 989-51-5; Gallocatechin gallate/GCG,4233-96-9; Epicatechin gallate/ECG, 1257-08-5, Yuanye, http://www.shyuanye.com/).

### Transient expression and GUS assays

The native promoter (2023 bp upstream sequence of the coding region) of *CsMYB5b* (KY827397) was isolated from ‘Shuchazao’ and inserted into pBI121 to replace the CaMV35S promoter followed by the GUS reporter. Similarly, the *CsMYB5a* (KY827396) promoter (2216 bp), the *CsANR* (GU992402) promoter (2179 bp), and the *CsLAR* (GU992401.1) promoter (2598 bp) were isolated and cloned to construct *CsMYB5a-Pro::GUS*, *CsANR-Pro::GUS*, and *CsLAR-Pro::GUS*, respectively. Six-week-old *N. tabacum* leaves were injected with either a reporter construct alone or combined with *35S::CsGLK1* or *35S::CsGLK2*. The constitutive expression vector (*35S::GUS*) was used as the positive control by *A. tumefaciens*-mediated transient expression. The methods of GUS staining and enzyme activity determination were as described previously [66].

### Electrophoretic mobility shift assay

The full-length coding sequences of *CsGLK1* and *CsGLK2* were amplified and inserted into the pET32a vector to generate the recombinant His-CsGLK1/CsGLK2 plasmid. The recombinant plasmid was introduced into *Rosetta* cells, and His-GLK1 and His-GLK2 proteins were expressed and purified using His Sepharose beads. Two conserved *cis*-element motifs of *CsMYB5b* promoters were found. The oligonucleotide probes containing GLK-binding sites CCAAAC and G-box TACGTT were labeled with biotin at the 3′ end of the sense strand according to the EMSA probe biotin labeling kit (Beyotime GS008). The EMSA was performed according to a previous study [67]. To confirm the specificity of the shifted band, a 100- to 200-fold amount of non-labeled cold probe was used. A Chemiluminescent EMSA Kit (Beyotime GS009) was used to detect the binding of protein–DNA.

### Gene suppression of *CsGLK1* and *CsGLK2* in tea plants

SOLIGO software (https://sfold.wadsworth.org/cgi-bin/soligo.pl) was used to select candidate AsODNs against *CsGLK1* and *CsGLK2* (Supplementary Data Table S3). AsODNs were synthesized by General Biosystems Company. Tea seedlings at the stage of one bud and two leaves were injected with 20 μM AsODN solution and treated for 24 hours (16 h light/8 h dark). Sense oligonucleotides (sODNs) were used as control. The experiments were performed according to the methods described in the previous study [43].

## Acknowledgements

This work was supported by grants from the National Natural Science Foundation of China (31900257 and 31972474) and Leading Talent Group Funding of Anhui Province (WRMR-2020-75).

## Author contributions

L.W., Y.L., and S.W. conceived and designed the experiments. L.W., X.T., S.Z., X.X., and M.L. performed all experiments and analyzed the data. S.W. and Y.L. wrote the manuscript. All authors approved the manuscript.

## Data availability

All data generated from the study appear in the submitted article.

## Conflict of interest

The authors declare no conflicts of interest.

## Supplementary data

Supplementary data is available at *Horticulture Research* online.

## Supplementary Material

Web_Material_uhac117
