# Supplementary material for: Tea GOLDEN2-LIKE genes enhance catechin biosynthesis through activating R2R3-MYB transcription factor
Source: Hortic Res. 2022 May 17;9:uhac117. doi: 10.1093/hr/uhac117 (PMC9347013; doi:10.1093/hr/uhac117)
Supplement: Web_Material_uhac117 [file web_material_uhac117.zip › Supplemental Figure revised.pdf]

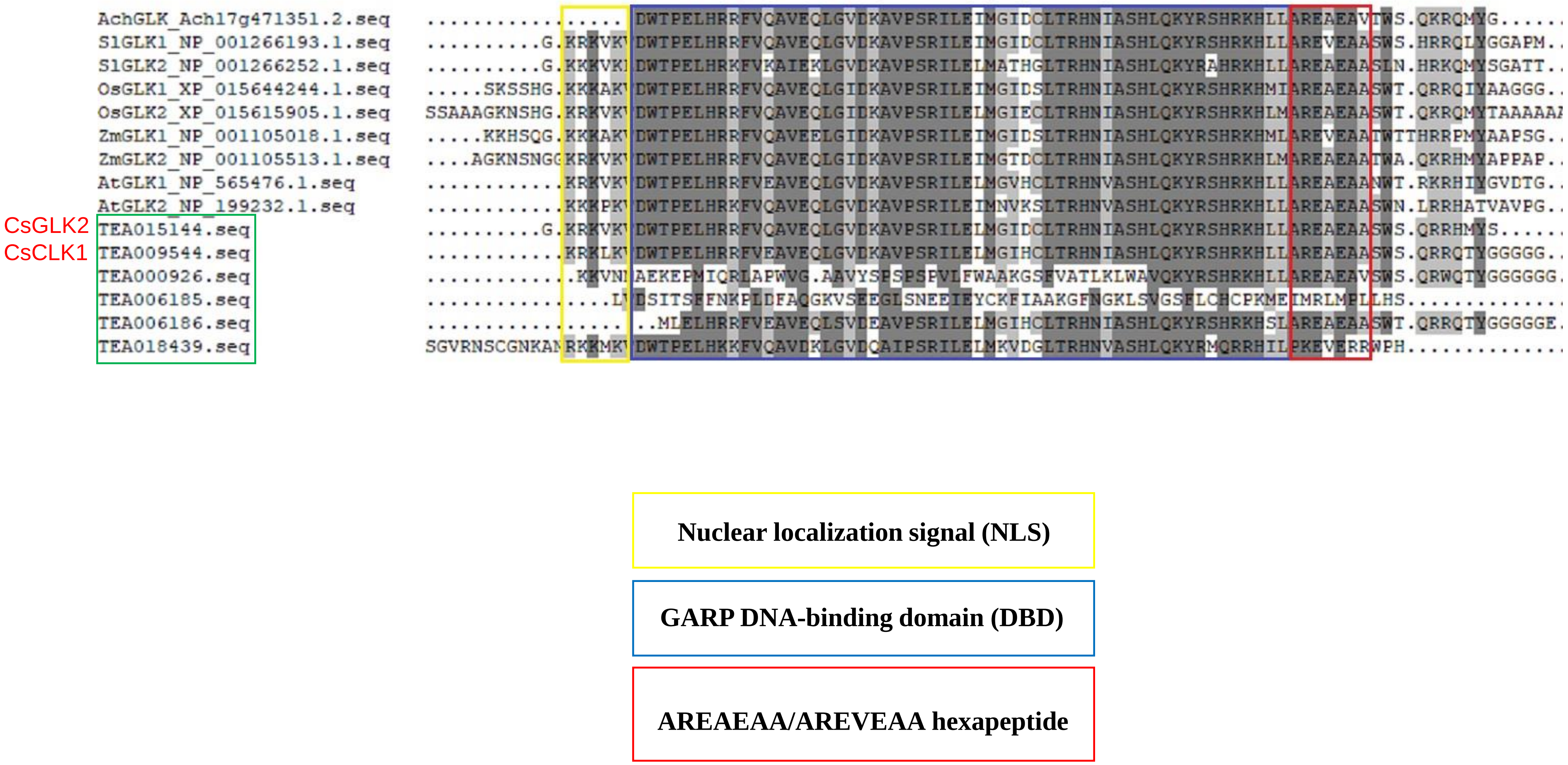

Figure S1. **Motif analysis of GLKs from different species including tea plants**

Motif alignment of CsGLK-like proteins including TEA015144 (named CsGLK2), TEA009544 (CsGLK1), TEA000926, TEA006185, TEA006196, and TEA018439 from *Camellia sinensis* with GLKs from other plant species. The conserved DBD domain motif are highlighted in a blue frame. The nuclear localization signals (NLS) at the upstream of the first helix were highlighted in a yellow frame. The AREAEAA/AREVEAA motifs at the downstream of the last α-helix were highlighted in a red frame.

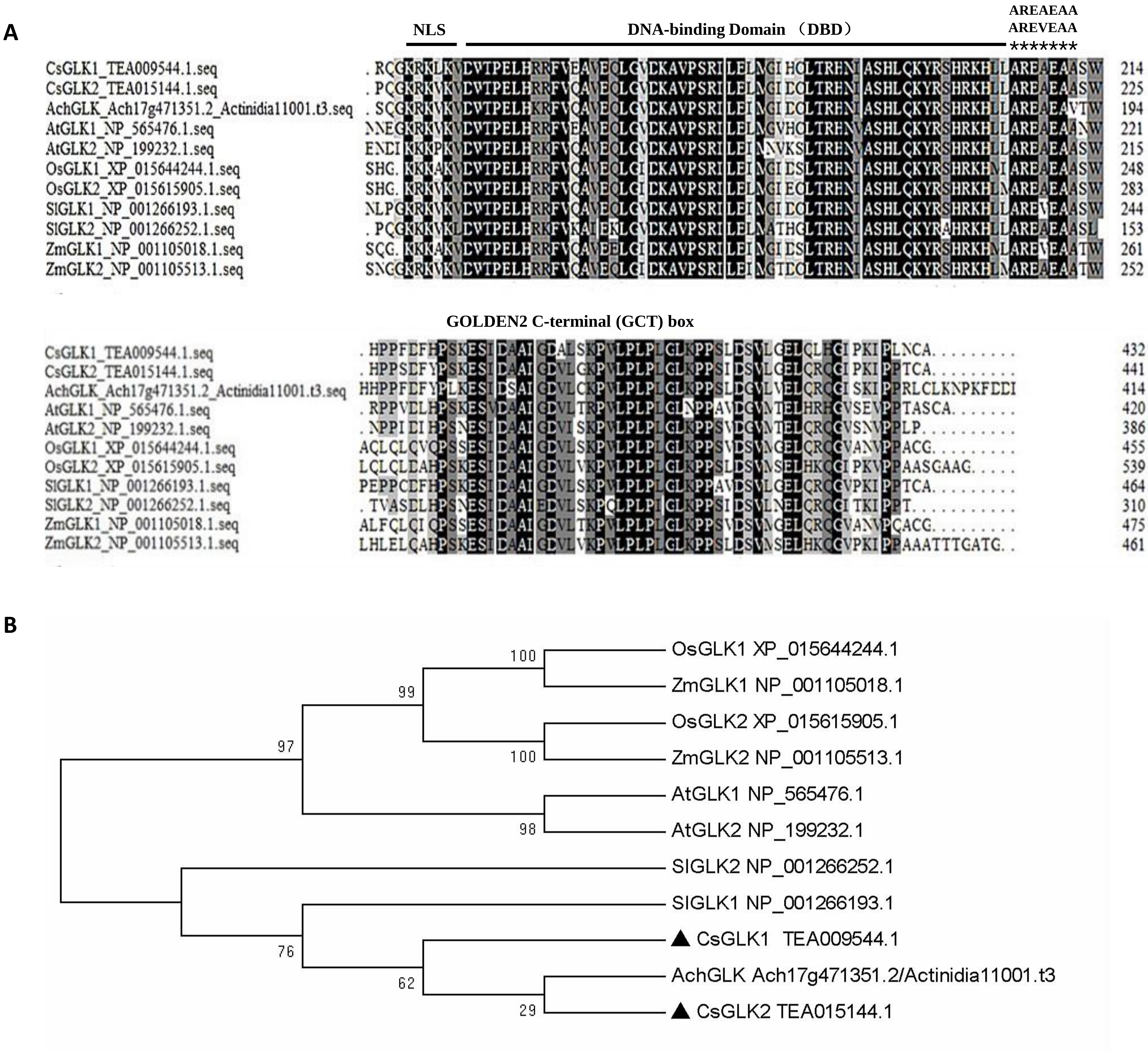

Figure S2. **Protein alignment and phylogenetic tree analysis of GLKs from different species**

Figure S2. Protein alignment and phylogenetic tree analysis of GLKs from different species

(A) Multiple alignment of the GLK amino acid sequences. The amino acid sequences of *GLK homologs from C. sinensis*, *Actinidia chinensis*, *Solanum lycopersicum*, *Oryza sativa*, *Arabidopsis thaliana* and *Zea mays* were aligned by the Clustal X. The identical residues are shaded in black with white letters. Residues with at least 75% conservation are shaded in deep gray with black letters. Residues with at least 50% conservation are shaded in gray with black letters.

(B) Phylogenetic tree of CsGLK1 and CsGLK2 (marked black triangles) and its homologous proteins from other plant species. Numbers beside the branches represent bootstrap values based on 1000 replicates.

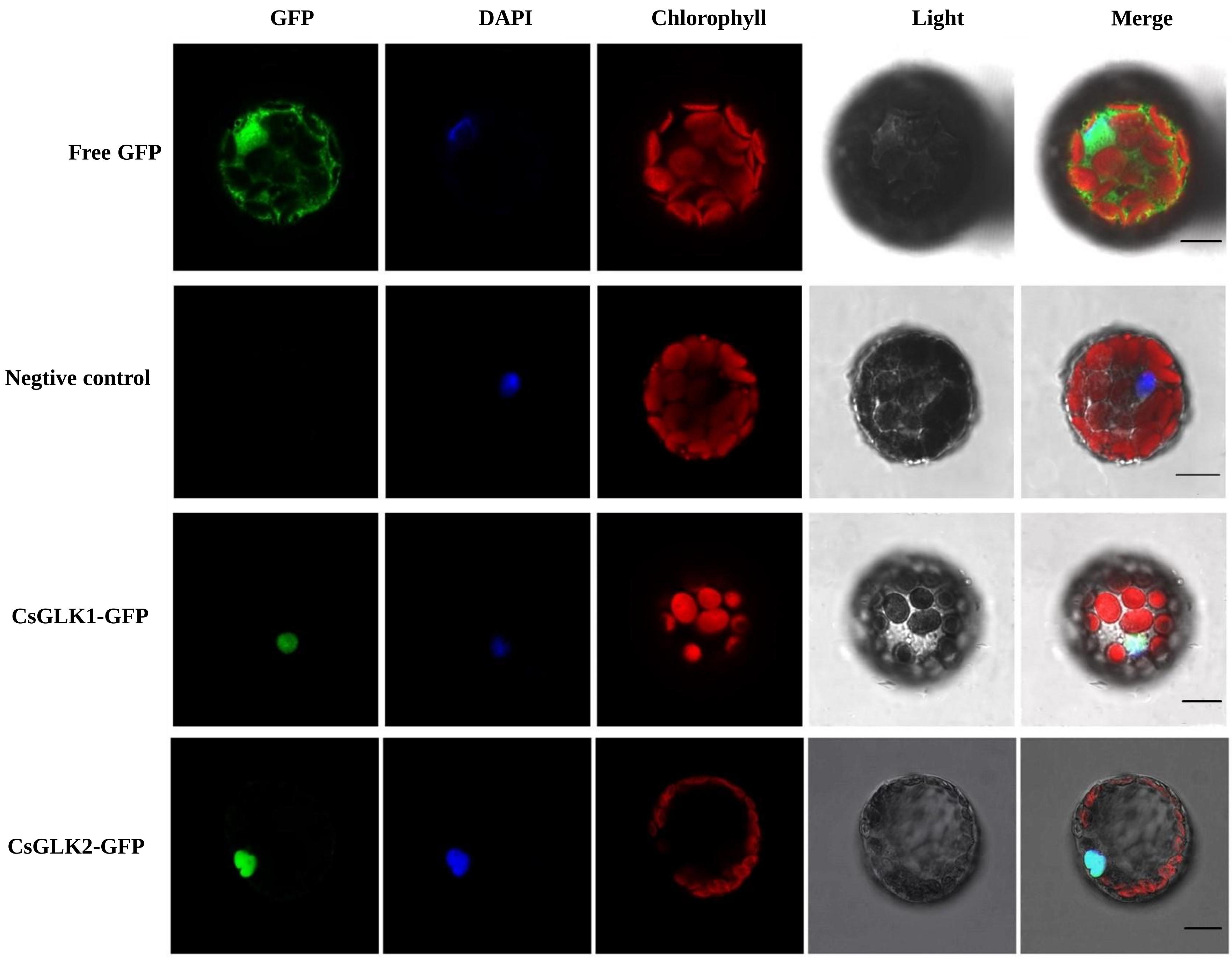

Figure S3. Subcellular localization of CsGLK1 and CsGLK2

CsGLK1-GFP and *CsGLK2-GFP* fusion genes was transiently expressed in *Nicotiana benthamiana* protoplasts. Free GFP and an untransformed plant protoplast served as positive and negative controls, respectively. Left to right: GFP, GFP fluorescence; DAPI, nucleus stained with DAPI; Chlorophyll, chlorophyll auto-fluorescence; Bright, a complete protoplast cell; Merge, combined fluorescence from GFP, DAPI, chlorophyll and bright fields. Scale bars=10 μm.

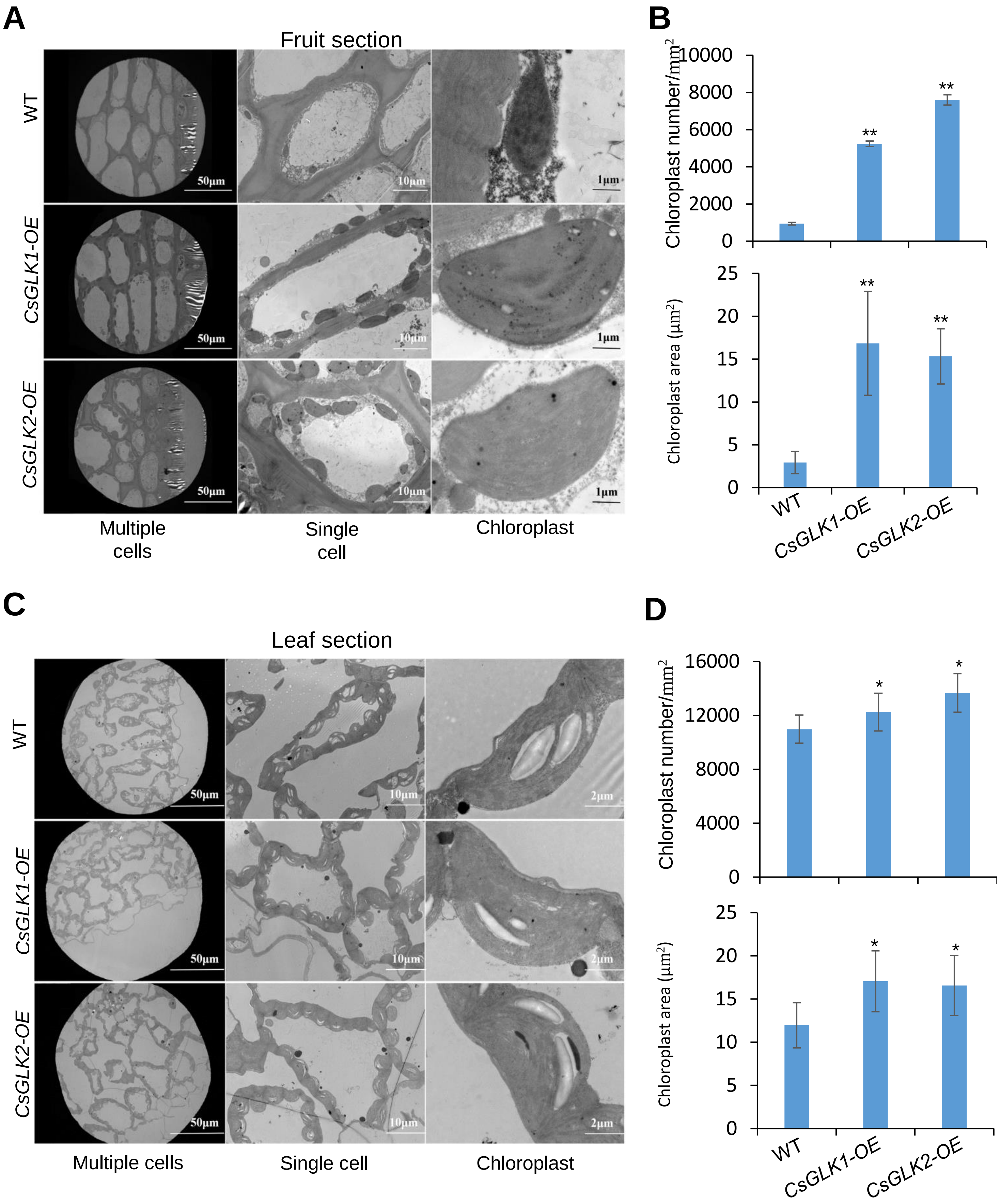

Figure S4. Overexpression of *CsGLK1* and *CsGLK2* enhance chloroplast development

Transmission electron microscopy (TEM) images of mature green fruit and leaf sections of WT, *CsGLK1*OE and *CsGLK2*OE plants. (A) The fruit tissues were fixed and sectioned at the same positions of the pericarps. (B) Number of chloroplasts per mm^2 was calculated and chloroplast area (μm^2) were measured according to the TEM images of fruit (A) by the software ImageJ. (C) The leaf tissues from 40-day-old plants were fixed and sectioned at the same positions. (D) Number of chloroplasts per mm^2 was calculated and chloroplast area (μm^2) were measured according to the TEM images of leaves (C) by the software ImageJ. Data represent average values ± SD (n>15) obtained from the three transgenic lines. "**" and "*" indicates the significant differences at P<0.001 and P<0.05 (Student t-test), respectively.

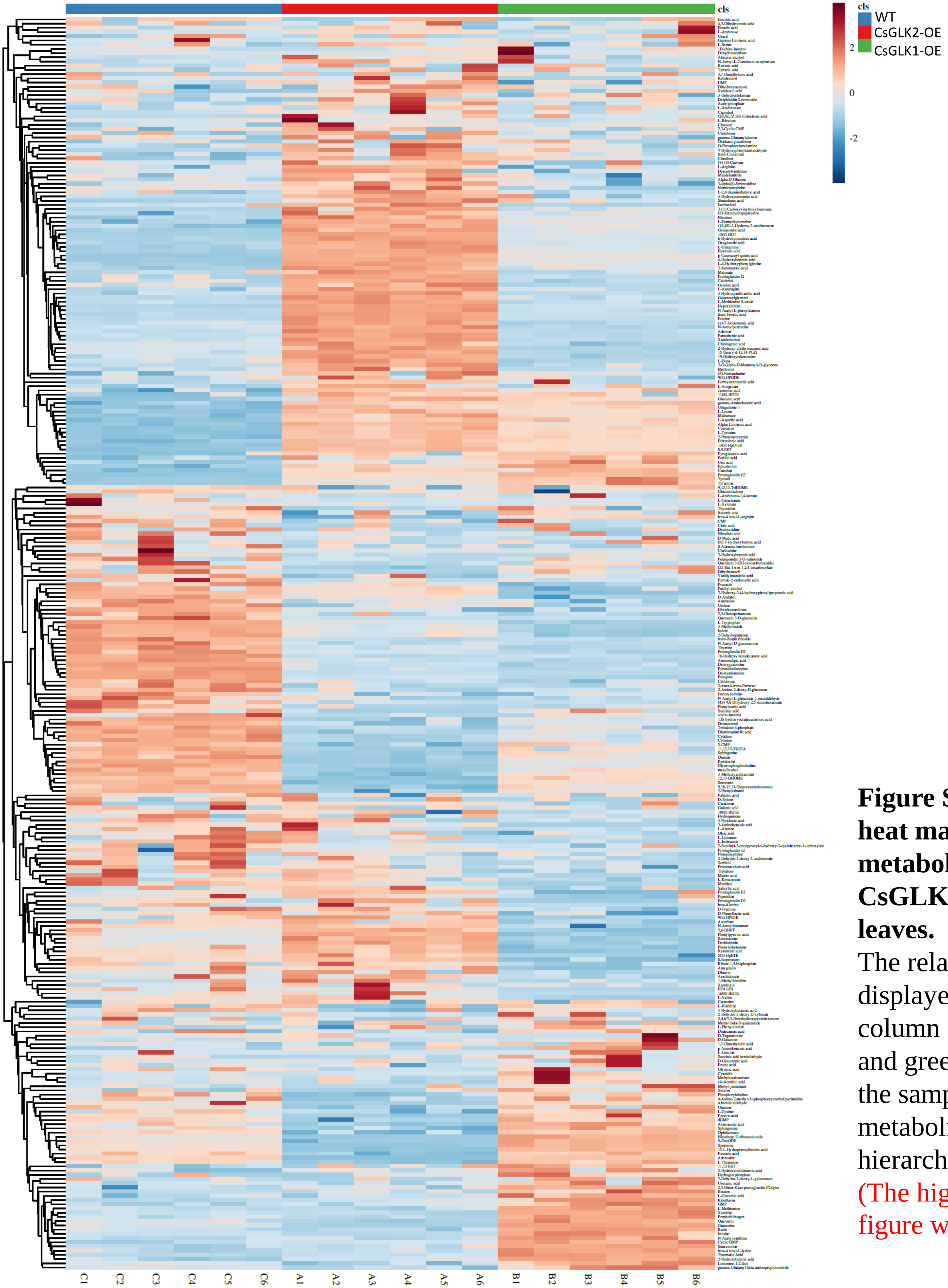

Figure S5. Hierarchical clustering heat map of all identified metabolites among WT and CsGLKs-overexpressing tomato leaves.

The relative content in the graph is displayed by color depth, in which the column (blue:WT, red:CsGLK2-OE, and green:CsGLK1-OE) represents the sample and the row represents the metabolite by agglomerate hierarchical clustering.

(The high-resolution version of this figure was uploaded in a separate file.)

A

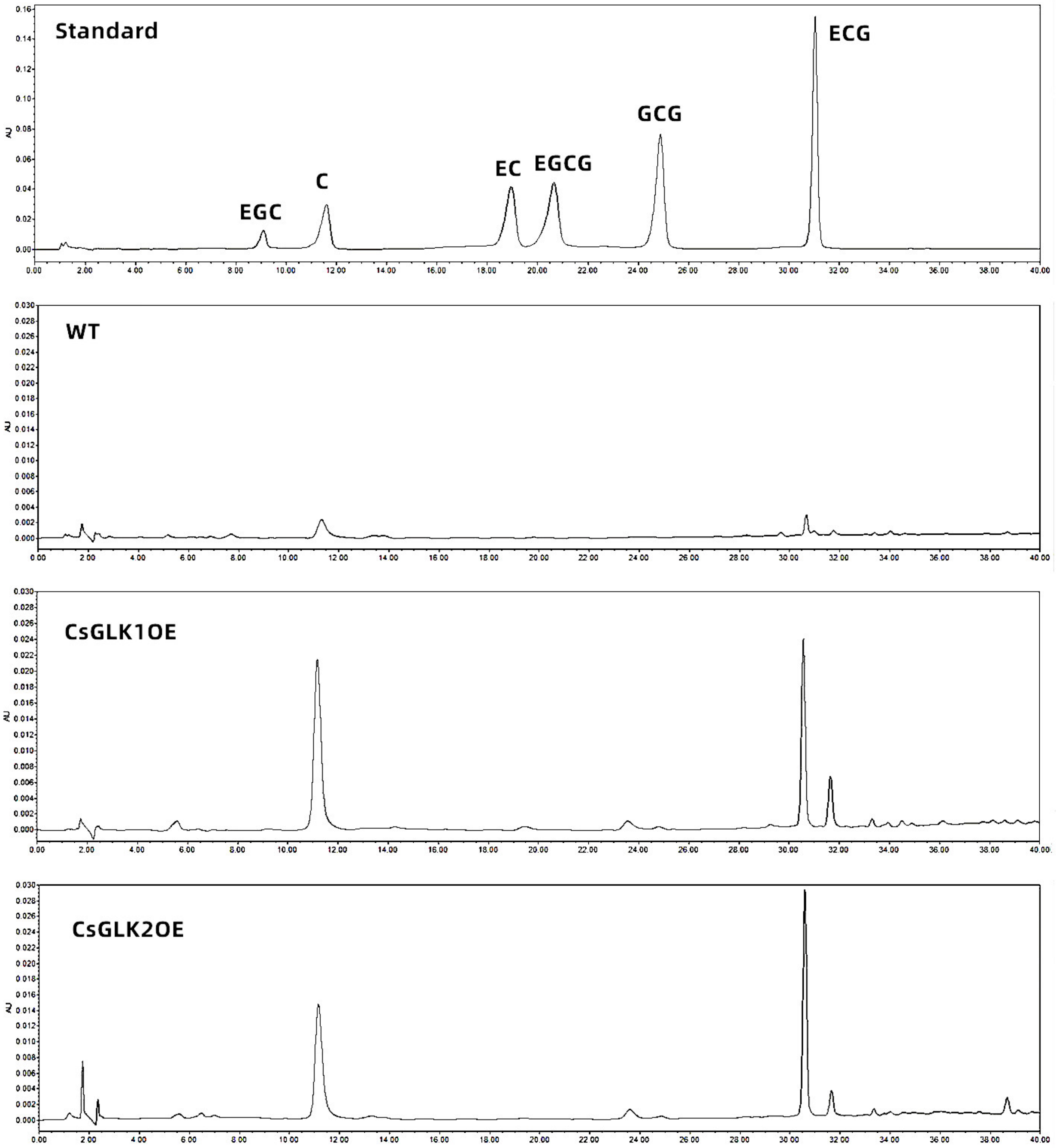

B

Sample Name	EGC	C	EC	EGCG	ECG
**WT**	NONE	0.00097±0.00002	NONE	NONE	0.00022±0.00007
**CsGLK1 OE**	NONE	0.22±0.04 * *	NONE	NONE	0.069±0.008 * *
**CsGLK2 OE**	NONE	0.16±0.02 * *	NONE	NONE	0.081±0.02 * *

Figure S6 HPLC analysis of catechin monomers in wild type and transgenic tomato plants

(**A**) Chromatogram of catechin monomers (epigallocatechin, EGC; catechin, C; epicatechin, EC; epigallocatechin gallate, EGCG; gallocatechin gallate, GCG; and epicatechin gallate, ECG) in leaves of wild type (WT) and GsGLKs-overexpressing (CsGLK1OE and CsGLK1OE) tomato plants. (**B**) Quantitative measurements of these catechin monomers (mg/g Fresh weight). All data represent the mean values±SD of three replicates. The asterisk indicates that the significant difference (**P < 0.01).

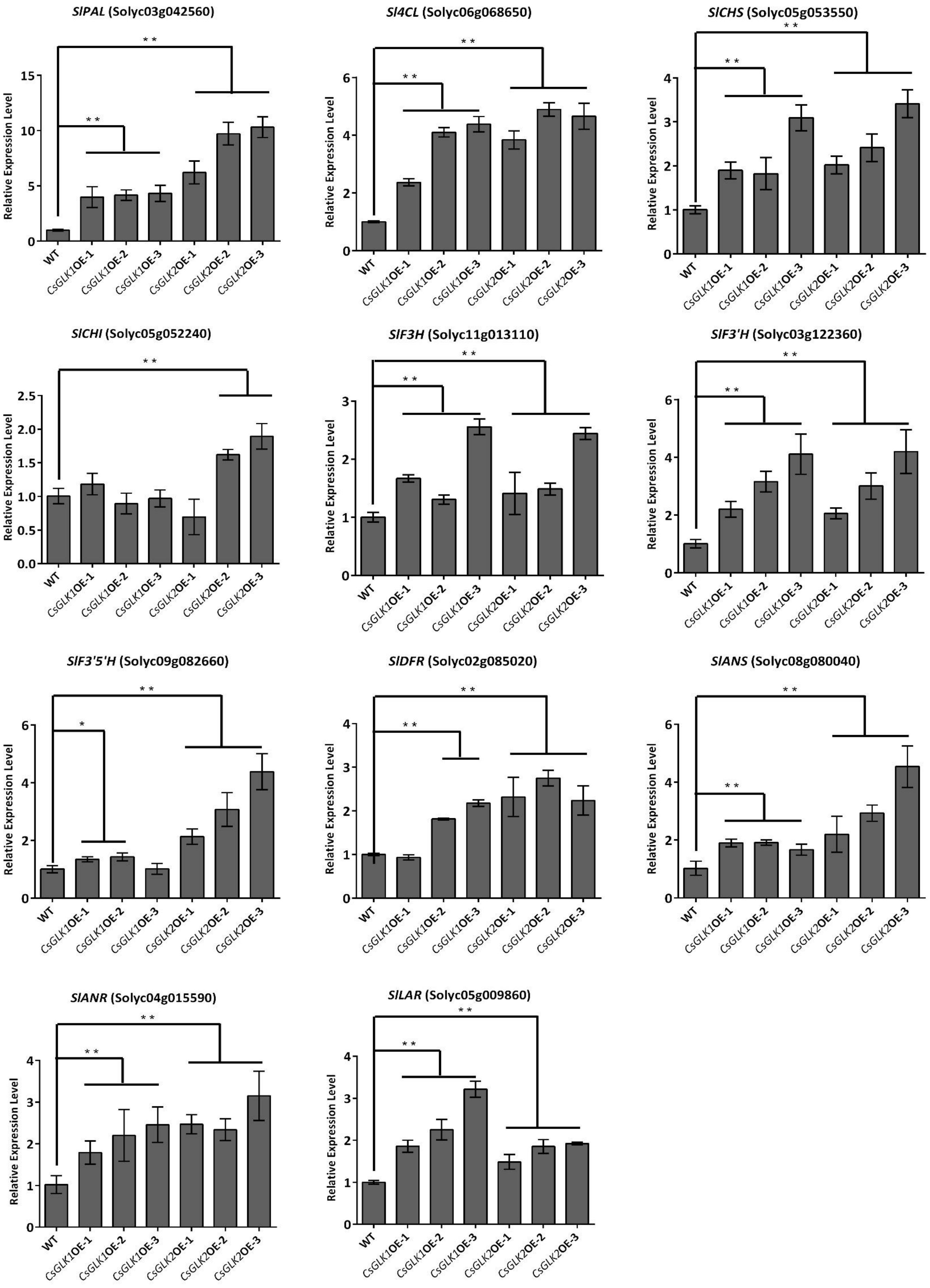

Figure S7. qRT-PCR validation of differentially expressed genes in flavonoid biosynthesis pathway. Validation of DEGs related to flavonoid biosynthesis among CsGLK1/2-OE and WT plants by qRT-PCR assays. The leaves from 40-day-old Micro-Tom plans were used for qRT-PCR analysis. Error bars represent standard deviation of three replicates. "**" and "*" indicates the significant differences at P<0.001 and P<0.05 (Student t-test), respectively.

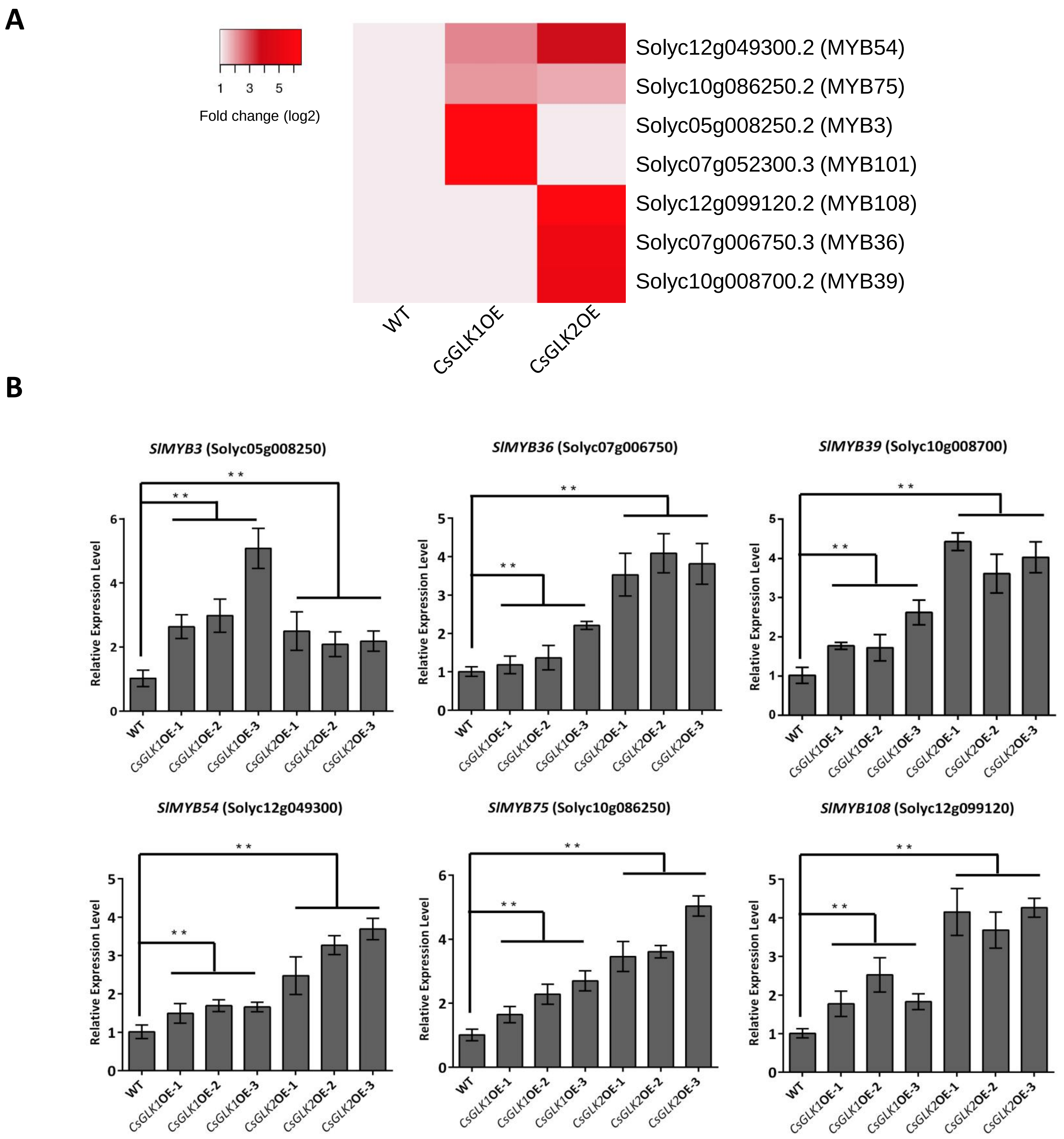

Figure S8 Heatmap of differentially expressed *SlMYB* genes and qRT-PCR validation

(A) Heatmap of differentially expressed SlMYB genes identified in the transcriptomic analysis of WT and CsGLKs-overexpressing tomato leaves. (B) Validation of up-regulated MYB genes between CsGLK1/2-OE and WT plants. The leaves from 40-day-old CsGLK1/2-OE and WT plants were used for qRT-PCR analysis. These six *MYB* genes were selected from correlation analysis of metabolomic and transcriptomic results. Error bars represent standard deviation of three replicates. "**" and "*" indicates the significant differences at P<0.01 and P<0.05 (Student t-test), respectively.

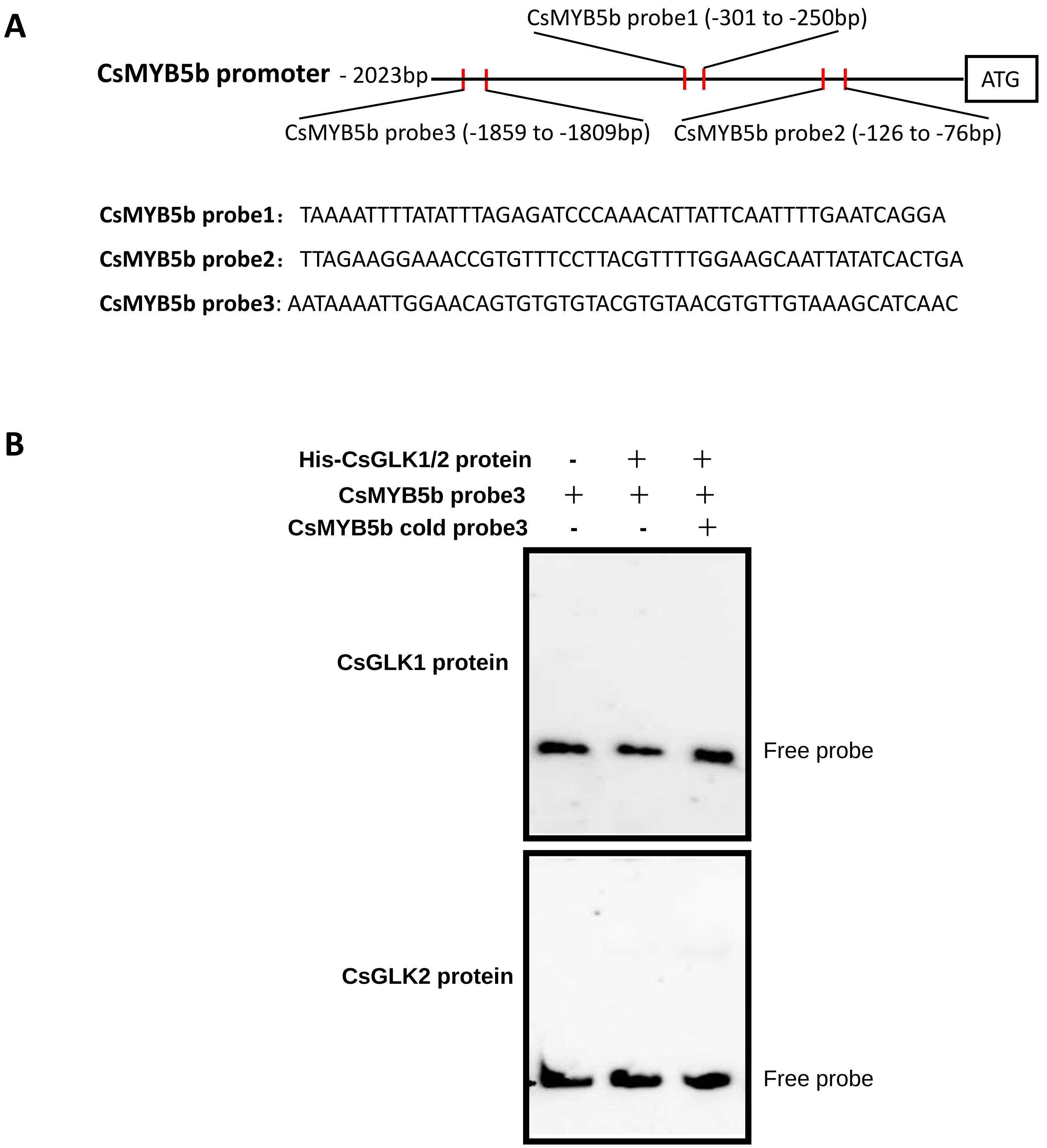

Figure S9 CsGLKs have no interaction with *CsMYB5b* probe3

(**A**) The sequence and locations of three probes (probe1, 2, and 3) in the promoter of *CsMYB5b*.

(**B**) EMSA showed no interaction between CsGLK1/2 and *CsMYB5b* probe3. Purified His-tagged CsGLK1and CsGLK2 protein was incubated with unlabeled probe (cold) or biotin-labeled probe, and DNA-protein complexes were separated on native polyacrylamide gels, then photographed. The presence or absence of specific probes is marked by the symbol ‘+’ or ‘–’.

A

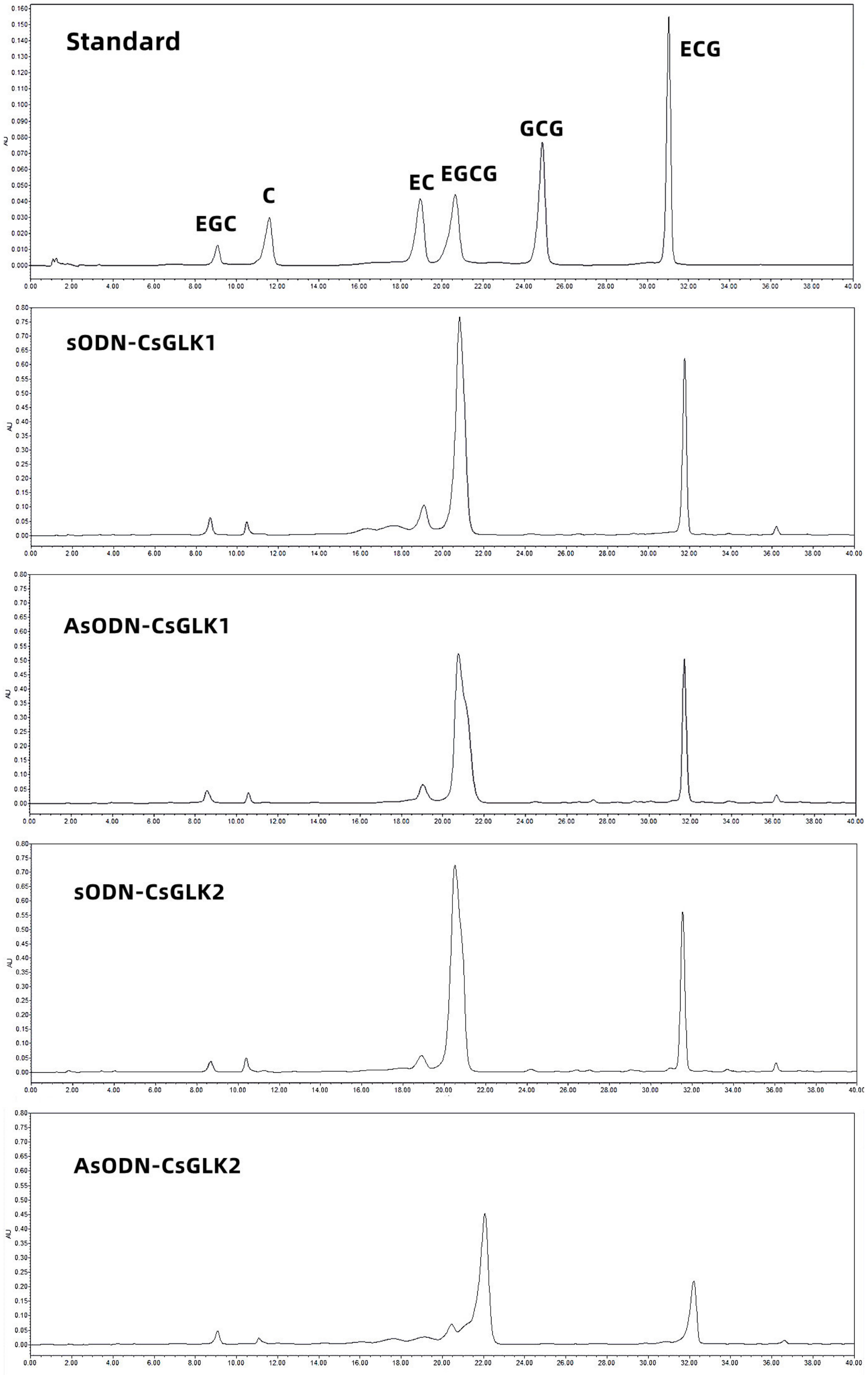

Figure S10 HPLC analysis of catechin monomers in tea leaves treated with sODN- or AsODN-CsGLKs

(**A**) Chromatogram of catechin monomers (epigallocatechin, EGC; catechin, C; epicatechin, EC; epigallocatechin gallate, EGCG; gallocatechin gallate, GCG; and epicatechin gallate, ECG) in tea leaves treated with sODN-CsGLK1, AsODN-CsGLK1, sODN-CsGLK2, and AsODN-CsGLK2. (**B**) Quantitative measurements of these catechin monomers (mg/g Fresh weight). All data represent the mean values±SD of three replicates. The asterisk indicates that the significant difference (*P<0.05, **P < 0.01).

B

Sample Name	EGC	C	EC	EGCG	ECG
sODN-CsGLK1	3.74±0.36	1.13±0.16	0.99±0.21	14.44±2.5	3.41±0.47
AsODN-CsGLK1	3.41±0.25 *	0.81±0.15 * *	0.85±0.12 *	8.1±0.74 * *	2.33±0.33 * *
sODN-CsGLK2	4.14±0.14	1.07±0.13	0.94±0.22	11.95±1.06	2.66±0.31
AsODN-CsGLK2	3.97±0.19 *	0.92±0.11 *	0.77±0.15 * *	6.57±0.84 * *	1.24±0.22 * *

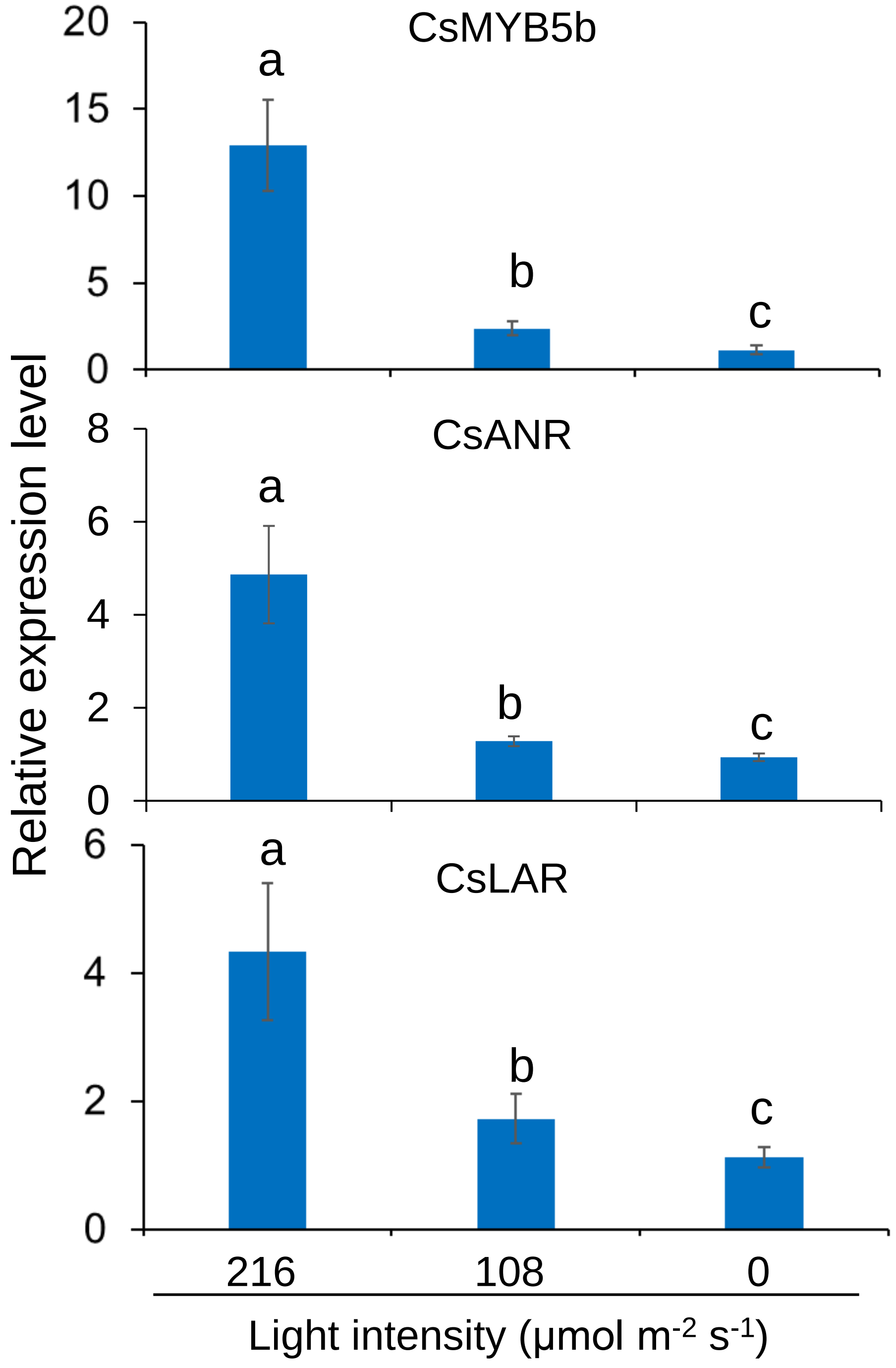

Figure S11 Light intensity affects expression levels of *CsMYB5b*, *CsANR* and *CsLAR*

The qRT-PCR assays of transcript levels of *CsMYB5b*, *CsANR*, and *CsLAR* in tea plants cultured in control light (light intensity: 216 μmol m^{-2} s^{-1}), shading (108 μmol m^{-2} s^{-1}) and dark (0 μmol m^{-2} s^{-1}) conditions for 24h. Error bars represent standard deviations of three biological replicates. The letters on the columns indicate statistically significant differences (Duncan test, P<0.05)

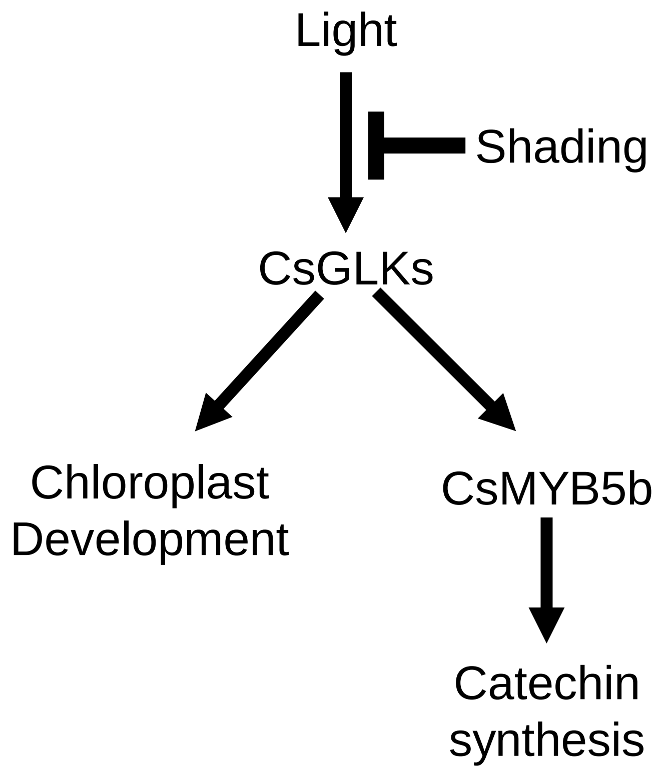

Figure S12 Schematic model of CsGLKs involved in light-induced catechin synthesis.
Light induces the expression of CsGLKs. On one hand, CsGLKs can promote chloroplast development; On the other hand, CsGLKs bind the promoter of *CsMYB5b* and enhance its expression and catechin synthesis. Shading treatment decreases expression levels of CsGLKs and thereby impairs catechin accumulation.
